# Nomogram for Predicting Recurrence-Free Survival in Chinese Women with Endometrial Cancer after Initial Therapy: External Validation

**DOI:** 10.1155/2020/2363545

**Published:** 2020-05-29

**Authors:** Yuan Cheng, Yangyang Dong, Wenjuan Tian, Hua Zhang, Xiaoping Li, Zhiqi Wang, Boer Shan, Yulan Ren, Lihui Wei, Huaying Wang, Jianliu Wang

**Affiliations:** ^1^Department of Obstetrics and Gynecology, Peking University People's Hospital, No. 11 Xizhimen South Street, Xicheng Dist., Beijing 100044, China; ^2^Department of Gynecology, Fudan University Shanghai Cancer Center, No. 255, Dong'An Road, Shanghai 200032, China; ^3^Research Center of Clinical Epidemiology, Peking University Third Hospital, Xueyuan Rd 38#, Haidian Dist., Beijing 100191, China

## Abstract

This study aimed at developing an available recurrence-free survival (RFS) model of endometrial cancer (EC) for accurate and individualized prognosis assessment. A training cohort of 520 women with EC who underwent initial surgical treatment and an external validation cohort of 445 eligible EC patients from 2006 to 2016 were analyzed retrospectively. Multivariable Cox proportional hazards regression models were used to develop nomograms for predicting recurrence. The concordance index (C-index) and the area under the receiver operating characteristic curve (AUC) were calculated to determine the discrimination of RFS prognostic scoring systems. Calibration plots were generated to examine the performance characteristics of the predictive nomograms. Regression analysis revealed that an advanced International Federation of Gynecology and Obstetrics (FIGO) stage, histological grade 3, primary tumor diameter ≥2 cm, and positive peritoneal cytology were independent prognostic factors for RFS in EC in the training set. The nomograms estimated RFS according to these four variables, with a C-index of 0.860, which was superior to that of FIGO stage (2009 criteria), at 0.809 (*P*=0.034), in the training cohort. Encouragingly, consistent results were observed in the validation set, with a C-index of 0.875 for the nomogram and a C-index of 0.833 for the FIGO staging (*P*=0.0137). Furthermore, the calibrations of the nomograms predicting 3- and 5-year RFS strongly corresponded to the actual survival outcome. In conclusion, this study developed an available nomogram with effective external validation and relatively appreciable discrimination and conformity for the accurate assessment of 3- and 5-year RFS in Chinese women with EC.

## 1. Introduction

Endometrial cancer (EC) is the most common gynecological malignancy, ranking as the fourth among female tumors in developed countries [1]. Epidemiological analysis in China showed that the morbidity and mortality rates of EC have increased over recent years [2, 3]. Endometrioid adenocarcinoma, also known as type I, is the most frequent histological subtype and accounts for about 65%. Nonendometrioid endometrial cancer which includes uterine serous carcinoma, clear cell cancer, and carcinosarcoma was identified as type II and accounts for about 35%. The 5-year overall survival of type I and type II was about 85% and 55%, respectively [4]. Although EC is detected early in most cases and patients begin receiving appropriate treatment with a good prognosis, the 5-year overall survival of patients with stage I–III EC ranges from 57% to 91% and with stage IV is 20–26% [5–7]. The prognosis of endometrial cancer is also affected by many other factors such as age, tumor grade, and positive peritoneal cytology [8]. Individual differences in recurrence or death in women with EC at 2 to 3 years after primary therapy vary widely [9, 10]. Hence, it is urgent to place greater emphasis on precise and individualized prognosis evaluation and monitoring strategies for the management of patients with EC.

Individualized mathematical nomograms have been widely adopted as auxiliary tools to guide clinical decision making in medical fields [11–14]. In 2014, AlHilli et al. developed nomograms stratified histologically to predict the overall survival of EC patients [10]. In 2016, a nomogram predicted a low recurrence rate in women with EC (stages I–III), which could reduce unnecessary treatment by 60% [15]. However, these risk-scoring models have only been performed in analyses of internal data, and they lack external validation in independent samples based on established mathematical formulas.

This study retrospectively analyzed data from 965 women with stage I–IV EC from two large-scale hospitals that have focused on EC treatment in China over the last 10 years. A nomogram with good discrimination and calibration was developed for both internal and external validation cohorts based on clinicopathological characteristics to predict the probability of 3- and 5-year recurrence-free survival (RFS) and overall survival in women with EC.

## 2. Materials and Methods

### 2.1. Patients

The retrospective cohort study included 965 patients who underwent hysterectomy for stage I–IV EC in Peking University People's Hospital (training cohort, *n* = 520) and Fudan University Shanghai Cancer Center (validation cohort, *n* = 445) from January 2006 to December 2016. The present study was approved by the Ethics Committee of Peking University People's Hospital and Fudan University Shanghai Cancer Center. The exclusion criteria were incomplete clinical data or lost to follow-up. The baseline characteristics collected for all patients were as follows: (1) essential variables: age and menopausal status; (2) clinical and surgical variables: surgical procedure (with or without lymphadenectomy); (3) pathological variables: FIGO stage, pathological type, differentiation status, tumor size, peritoneal cytology status, lymphovascular space involvement (LVSI), lymph node metastasis, depth of myometrial invasion, and cervical stromal invasion (clinical stage and histological grade for all patients were classified in accordance with the 2009 FIGO criteria and pathological type followed by the two types of endometrial carcinoma of Bokhman in 1983); and (4) adjuvant therapy information: radiotherapy, chemotherapy, or their combination. Endometrial cancer patients with high-risk factors were performed adjuvant treatment after surgery according to pathological findings and comprehensive multidisciplinary discussion based on international guidelines. Generally, high-risk factors usually include age ≥ 60, myometrial invasion ≥ 50%, grade 3, LVSI positive, and type II endometrial cancer. Patients without any high-risk factor were considered as in the low-risk group who just needed follow-up. Patients with high-risk factors (age ≥ 60, grade 3, myometrial invasion ≥ 50%, stage II, and LVSI) were recommended for radiotherapy or chemotherapy. If the patients with more high-risk factors (stage III-IV, type II endometrial cancer), they were undergone combination regimen (radiotherapy and chemotherapy).

### 2.2. Treatment and Follow-Up

All women with stage I–IV EC were enrolled if they had undergone initial surgical treatment, including total hysterectomy with bilateral salpingo-oophorectomy with or without systematic lymph node dissection (pelvic ± para-aortic lymphadenectomy). Patients who were at high risk for cancer development and those with an advanced cancer stage underwent postoperative adjuvant radiotherapy, systemic chemotherapy, or their combination. Patients were followed up after initial surgery. And the occurrence of recurrence or death of the patients was recorded. Physical examination and diagnostic imaging tests were performed according to the findings.

### 2.3. Statistical Analysis

#### 2.3.1. Definition of RFS

The clinical outcome was evaluated according to recurrence-free survival (RFS). The duration of follow-up for RFS was defined as the time from hysterectomy-based surgical treatment to the date of first recurrence or last follow-up if there was no recurrence. In addition, Kaplan–Meier cumulative survival probability was used in this study. Cumulative survival probability was calculated by multiplying probabilities for each prior relapse time [16].

#### 2.3.2. Nomogram of Prediction Model

The clinical and pathological variables were evaluated for an association with RFS by univariate and multivariate Cox proportional hazards regression analyses. Associations are represented by the hazard ratio (HR) and corresponding 95% confidence intervals (CIs) assessed from the model. Variables with *P* < 0.05 were identified as independent risk factors for RFS and were retained in the final model. Furthermore, the selected high-risk variables were included in the Cox proportional hazards models of RFS. The risk coefficient of each factor was calculated and included in the equations of the individual prediction models for each patient and is presented as nomograms.

#### 2.3.3. Validation of the Prediction Models

The discrimination ability of the prediction models was estimated using the Harrell C-index. The C-index was calculated by Cox regression models of 1000 random bootstrap resamples with the same sample size for assessing model accuracy [17]. The C-index ranges from 0.5 to 1, with greater than 0.5 defined as having predictive power. Kaplan–Meier curves were plotted according to the bisection method for stratified management by the nomogram scores for the high- and low-risk groups. Calibration plots were examined by graphic charts for monitoring the average and maximal errors between the predicted 3- and 5-year probability of RFS and the actual outcome frequencies by the Kaplan–Meier method. The specificity and sensitivity of the models based on the nomogram compared with the 2009 FIGO stage for predicting RFS were evaluated by calculating the area under the receiver operating characteristic (ROC) curve (AUC).

#### 2.3.4. Additional Statistical Analysis

The follow-up time was described using median, ranging from min to max; frequencies and proportions were used for categorical variables. The clinical features of the cohorts were analyzed using Student's *t*-test. *P* < 0.05 was considered statistically significant. Data were collected using Microsoft Excel and converted to .sav files. All analyses were performed using SPSS v20.0 and R v2.15.0 with the Hmisc, rms, and Presence Absence packages.

## 3. Results

### 3.1. Training Cohort

**Figure 1:**
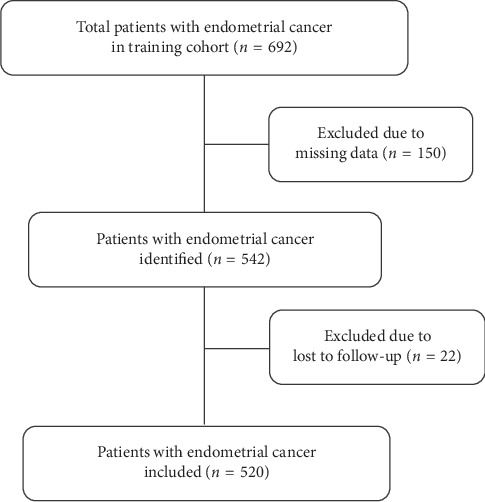
Flow diagram of the study participants.

We included 520 women with EC in the training cohort (Figure 1). The percentage of types I EC was 87.5%. The distribution of women EC was 84.8% with stages I and II and 15.2% with stages III and stage IV. The number of women with low-grade and high-grade EC was 393 (75.6%) and 127 (24.4%), respectively. The clinicopathological characteristics of women are shown in Table 1. The median follow-up period for RFS was 53 months (range, 1–110); 46 women (8.8%) relapsed, and 474 (91.2%) showed no recurrence. The median time from initial therapy to recurrence was 12 months (range, 1–100). The mean (SD) 3- and 5-year RFS was 92.0% ± 1.3% and 90.1% ± 1.6%, respectively.

### 3.2. Prediction Nomogram

**Figure 2:**
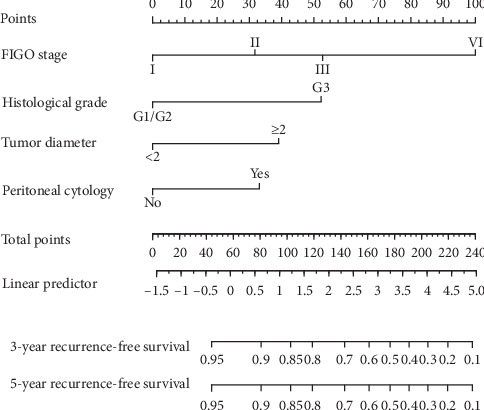
Nomograms for predicting 3- and 5-year recurrence-free survival (RFS) in patients with endometrial cancer. In order to evaluate the recurrence-free survival rate of each patient, the score of each variable was calculated by the value of the “Points” axis, and the sum of the values of all variables was corresponding to the number of the “Total points” axis. The vertical line of the total score was corresponding to 3- and 5-year probability of recurrence-free survival.

The clinicopathological characteristics of EC patients from the training sets with or without recurrence were analyzed (Supplementary Table 1). The results of the univariate and multivariate analyses revealed that four of the screened variables including advanced stage, G3, primary tumor diameter ≥ 2 cm, and positive peritoneal cytology were independent prognostic factors in the training group (Table 2). The predictive nomograms were constructed based on the selected covariates to assess the probability of 3- and 5-year RFS in the training set (Figure 2). The incorporated mathematical formula of the nomograms involved FIGO stages II (HR = 2.4; 95% CI: 0.9–6.7), III (HR = 4.2; 95% CI: 1.9–9.5), and IV (HR = 15.1; 95% CI: 5.4–42.8), G3 (HR = 4.2; 95% CI: 2.1–8.4), tumor diameter ≥ 2 (HR = 2.9; 95% CI: 1.0–8.4), and positive peritoneal cytology (HR = 2.5; 95% CI: 1.2–5.0), with further score transformation. According to the formulas for the nomograms, the total scores for each patient for 3- and 5-year RFS could be easily and accurately calculated to individualize the prognosis.

### 3.3. Comparison with FIGO Stage

**Figure 3:**
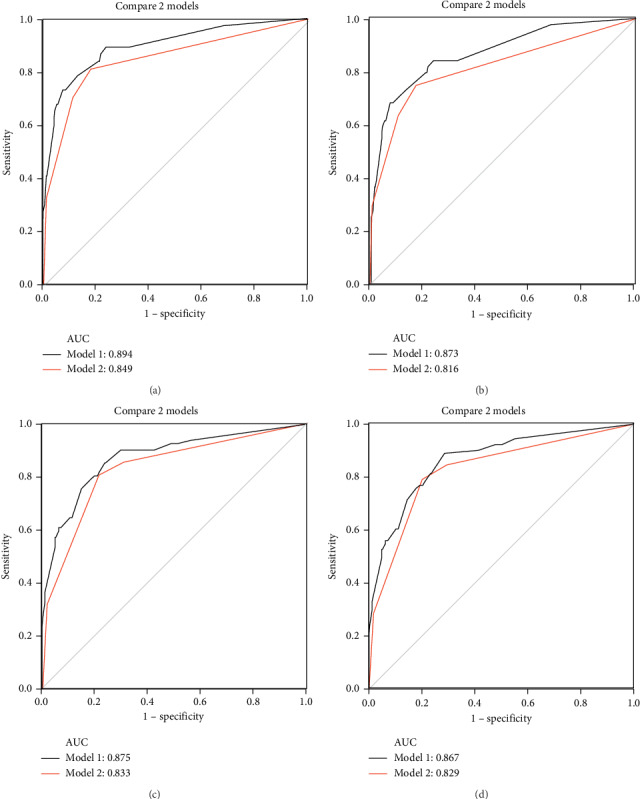
Area under the receiver operating characteristic curve (AUC) values of the nomogram and the 2009 International Federation of Gynecology and Obstetrics (FIGO) stage system for the training and validation cohorts to assess the 3- and 5-year recurrence-free survival (RFS). (a, b) Comparing the AUCs of nomogram with FIGO stage in the training group (T) for predicting 3- and 5-year recurrence-free survival (RFS) in patients with endometrial cancer. (c, d) Comparing the AUCs of nomogram with FIGO stage in the validation group (V) for predicting 3- and 5-year recurrence-free survival (RFS) in patients with endometrial cancer. Model 1 of black line represents nomogram; model 2 of red line represents FIGO stage. (a) T: 3 RFS-AUC nomogram:FIGO, (b) T: 5 RFS-AUC nomogram:FIGO, (c) V: 3 RFS-AUC nomogram:FIGO, and (d) V: 5 RFS-AUC nomogram:FIGO.

The discrimination ability of the nomograms was compared with that of FIGO stage. The C-index of the RFS nomogram was 0.860 (95% CI: 0.797–0.923), which was superior to that of the 2009 FIGO classification, at 0.809 (95% CI: 0.738–0.879; *P*=0.034) in the training set. Furthermore, the AUCs for the 3- and 5-year RFS nomograms were 0.894 (95% CI: 0.832–0.956) and 0.873 (95% CI: 0.812–0.934), respectively, which was superior to that of the 2009 FIGO classification at 0.849 (95% CI: 0.777–0.920; *P*=0.0268) and 0.816 (95% CI: 0.75–0.89; *P*=0.0037), respectively (Figures 3(a) and 3(b)).

### 3.4. Validation of the Nomogram

We recruited 445 eligible women with EC for the validation cohort. The clinicopathological characteristics of EC patients from the validation cohort with or without recurrence were analyzed (Supplementary Tables 2–4). The frequency of type I and type II EC was 359 (80.7%) and 86 (19.3%), respectively. The distribution of women with EC was as follows: 264 (59.3%) with stage I, 38 (8.5%) with stage II, 111 (25.0%) with stage III, and 32 (7.2%) with stage IV. The number of women with low-grade and high-grade EC was 297 (66.7%) and 148 (33.3%), respectively. The median follow-up period for RFS was 28 months (range, 1–112); 92 (20.7%) women relapsed, and 353 (79.3%) showed no recurrence. The median time from initial therapy to recurrence was 12 months (range, 1–63). The mean (SD) 3- and 5-year RFS was 78.8% ± 1.2% and 72.8% ± 1.6%, respectively.

The 3- and 5-year RFS rates were also calculated in the validation cohort based on the nomogram for the training cohort. The C-index for the RFS nomogram was 0.875 (95% CI: 0.829–0.921), which was superior to that of the 2009 FIGO classification at 0.833 (95% CI: 0.785–0.882; *P*=0.0137). The AUCs for 3- and 5-year RFS were 0.875 (95% CI: 0.829–0.921) and 0.867 (95% CI: 0.823–0.910), respectively, which were superior to those of the 2009 FIGO classification at 0.833 (95% CI: 0.785–0.882; *P*=0.0137) and 0.829 (95% CI: 0.785–0.882; *P*=0.0296), respectively (Figures 3(c) and 3(d)).

**Figure 4:**
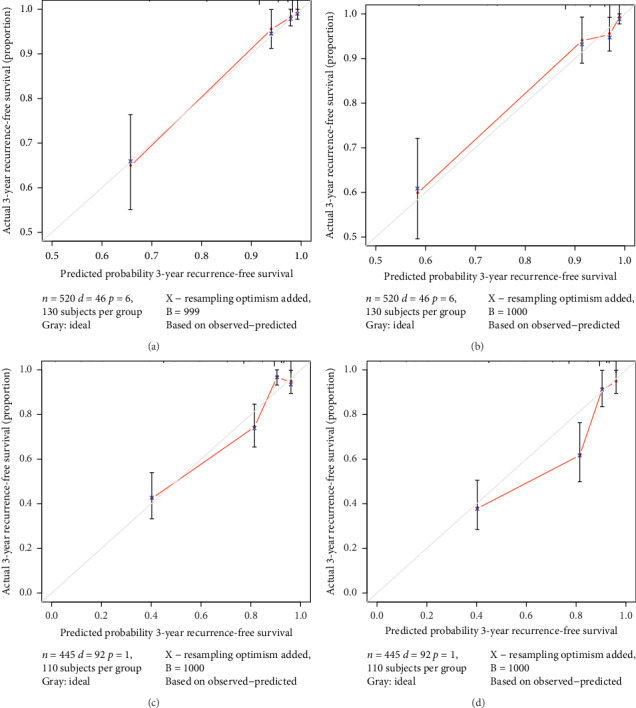
Calibration of the nomograms for 3- and 5-year recurrence-free survival (RFS) in patients with stage I–IV endometrial cancer in the training and validation cohorts. *X*-axis indicates the predicting probability of nomogram. *Y*-axis shows the actual 3- or 5-year probability of survival as assessed by Kaplan–Meier curves. Red line represents the predicted probability of nomogram. Gray line represents ideal consistency between the prediction and actual probabilities of 3- or 5-year RFS. Vertical bars represent 95% CI. Blue dots correspond to the accuracy of the prediction. (a, b) Calibrations of the nomogram in the training cohort for predicting 3- and 5-year RFS. (c, d) Calibrations of the nomogram in the validation cohort for predicting 3- and 5-year RFS.

Calibration plots for the nomograms to predict 3- and 5-year RFS were calculated in the internal validation. The predicted 3- and 5-year RFS rates were similar to the actual survival rates, with small average error rates of less than 10% and a lack of bias, as represented by the dotted lines in Figures 4(a) and 4(b). Moreover, external validation showed no dramatic differences between the predicted and actual 3- and 5-year RFS rates (Figures 4(c) and 4(d)).

### 3.5. Optimal Nomogram Threshold and Redistribution

The low- and high-risk groups were defined according to the optimal threshold of the ROC calculated from the recurrence distribution of each probability of the RFS nomograms in the training cohort (*P*=0.029). We further analyzed the distribution of patients in the low- and high-risk groups estimated by the nomogram scores. The frequency of low- and high-risk EC was 371 (71.3%) and 149 (28.7%), respectively (Table 3). We found individual differences in recurrence in women with EC after redistribution. In the low-risk group, the characteristic distribution among EC patients with high-risk factors was as follows: advanced stage (4, 1.1%), grade 3 (22, 5.9%), primary tumor diameter ≥ 2 cm (192, 51.8%), and positive peritoneal cytology (1, 0.3%). In the high-risk group, the number of patients with low-risk factors was as follows: FIGO stage I, G1/G2, primary tumor diameter < 2 cm, and negative peritoneal cytology was 55 (36.9%), 44 (29.5%), 10 (6.7%), and 103 (69.1%), respectively.

## 4. Discussion

Predictive nomograms for the assessment of EC prognosis and recurrence have preliminarily been well developed in Europe and the United States [10, 15]. Nevertheless, these prediction models are heterogeneous according to women presenting with EC in different populations [18]. As far as we know, a nomogram to predict recurrence in EC patients based on the Chinese population has not been established until now. In the current study, we developed a nomogram to predict recurrence in women with EC in China, which was well validated in an independent cohort from Fudan University Shanghai Cancer Center. An available nomogram for predicting RFS in Chinese women with EC after initial therapy was preliminarily developed and externally validated. We have sorted out a table to compare the predicting recurrence-free survival model with previous studies (Table 4). (1) The predictive model was established based on the data from patients with endometrial cancer in all stages (I–IV), not just focusing on early stages (I–III) in this study. (2) The independent risk factors of endometrial cancer recurrence selected by multivariate analysis in the present study were some differences from those in the previous studies. Advanced stage, grade 3, primary tumor diameter ≥ 2 cm, and positive peritoneal cytology were independent recurrent factors in the training group in this study. One or more of these indicators have been included in previously established models; however, the combination of these four indicators was included in a recurrence model for the first time in this study. (3) Other studies have only predicted 3-year relapse-free survival in patients with endometrial cancer. Our prediction model is focused on both 3-year relapse-free survival and 5-year relapse-free survival. The model established in this study proved to be accurate and stable through external verification. The accuracy and the verification of the model were higher than those of other types of models. (4) We also compared our prediction model with the FIGO staging and found that it was superior to the FIGO staging in the prediction of 3-year or 5-year relapse-free survival of patients with endometrial cancer. This comparison has not been performed in previous studies.

Four independent risk factors, i.e., FIGO stage, histological grade, tumor diameter, and peritoneal cytology status, were primarily deemed predictive factors of RFS to develop a nomogram for patients with stage I–IV EC in the training set after confounding factors adjustment. 455 cases of type I and 65 cases of type II were included in this study. However, pathological type was not selected as an independent risk factor for recurrence in patients with endometrial cancer. Accumulating evidence has affirmed that LVSI significantly contributes to replapse in stage (I–III) and high-risk EC patients [9, 14]. However, LVSI was not the most appropriate pathological variable for assessing recurrence risk in our study, which may be due to the limited sample size.

The influence of the peritoneal cytology status on the prognosis of women with EC remains and should be further evaluated. Previous multivariate analyses have revealed that positive peritoneal cytology could predict relapse and tumor-related death in early-stage EC [19–23]. However, other studies found that, in low-risk patients with EC, positive peritoneal cytology did not affect the 5-year disease-free survival rate [24, 25]. Positive peritoneal cytology has also previously shown no effects on overall survival or disease-free survival in patients with low- or intermediate-risk disease [26, 27]. In the present study, we found that positive peritoneal cytology was a critical and independent prognostic factor of EC recurrence in both cohorts. The possible reason may be that our study involved EC patients with progressive staging or poor differentiation.

Notably, the results showed that the RFS estimation of the nomograms according to the four variables was superior to that of the 2009 FIGO classification in the training cohort. The calibrations of the nomograms predicting 3- and 5-year RFS highly corresponded to the actual survival rates, with minute average error rates of less than 10% for both. We further divided the patients into low- and high-risk groups according to the optimal threshold of ROC from the recurrence distribution of each probability of the RFS nomograms. Predictably, we discovered women presenting only one high-risk factor who were represented in the low-risk group and women with low-stage disease presenting other high-risk factors allocated to the high-risk group. Therefore, individual differences in prognosis are more common in EC patients.

Kondalsamy-Chennakesavan et al. developed nomograms to predict EC recurrence in 2097 patients with stage I–III EC from 1997 to 2009. The multivariate Cox model indicated that age, FIGO stage, histological grade, LVSI, tumor type, and peritoneal cytology status were independent prognostic factors of EC relapse [28]. Bendifallah et al. attempted to validate the Kondalsamy-Chennakesavan nomogram of EC for prognosis evaluation in 271 cases with stage I–III EC using an independent, multicenter external patient cohort. However, results showed that the nomogram was only partially generalized in another independent population, with a discrimination ability of 0.66 for 3-year recurrence-free survival [18]. The study reported disparate population characteristics, recommendations for lymph node resection, and adjuvant chemotherapy in multiple regions, and the efficacy of the prediction model in the external validation was relatively limited. Further optimization and improvement of nomograms and existing risk stratification strategies are needed [29]. In the present study, based on the four variables of the model, we verified the accuracy of our nomogram in an external cohort, with a C-index of 0.875 for the predicted 3-year RFS of EC. Additionally, the calibration curve was acceptable despite little differences in several clinical characteristics, recurrence outcomes, and follow-up times between the two populations. The number of patients with advanced endometrial cancer and the proportion of patients with recurrence in the validation cohort were higher than those in the training cohort. However, the follow-up time of EC patients in the validation cohort was shorter. More optimistically, the above four covariates used to develop the prediction models in the training cohort were also identified as independent prognostic factors of EC relapse in the validation cohort by multivariate analysis.

Our study has some limitations. Retrospective clinical data with uncertain potential confounding factors could negatively affect the accuracy of the results. Inevitably, many observations with missing data were deleted, which could have caused bias. Abu-Rustum et al. included the number of lymph nodes removed during comprehensive surgery in a predictive model of prognosis for women with EC [14]. AlHilli et al. revealed inadequate/negative lymphadenectomy as an independent risk factor in low-risk patients with EC [10]. We also assessed the value of surgical treatment and adjuvant therapy in the recurrence of patients with EC. Univariate analysis showed that they were associated with endometrial cancer recurrence. However, multivariate analysis showed that they were not independent risk factors for endometrial cancer recurrence in our study. The reason may be the limited number of cases or other interaction factors. Additionally, the modeling and validation groups differed in clinical characteristics and recurrence rates, which might also have affected the results. Finally, further optimization of this model in a national multicenter study is needed.

We developed a clinically available and relatively precise model based on nomograms to predict RFS in Chinese women with EC. The study specifically incorporated the four independent prognosis covariates of advanced stage, grade 3, tumor diameter ≥2 cm, and positive peritoneal cytology and helped develop a tool that may be conducive for developing individualized therapeutic strategies for Chinese patients with EC.

## Figures and Tables

**Table 1:** The clinicopathological characteristics of the training cohort.

Variables	Training cohort, *n* = 520
	(no. of patients) (%)
Age (years)	
<60	339 (65.2)
≥60	181 (34.8)

Menopausal status	
No	177 (34.0)
Yes	343 (66.0)

Depth of myometrial invasion	
<50%	391 (75.2)
≥50%	129 (24.8)

Cervical stromal invasion	
No	461 (88.7)
Yes	59 (11.3)

Adnexal involvement	
No	492 (94.6)
Yes	28 (5.4)

FIGO stage	
I	404 (77.7)
II	37 (7.1)
III	62 (11.9)
IV	17 (3.3)

Histological grade	
Grade 1	161 (31.0)
Grade 2	232 (44.6)
Grade 3	127 (24.4)

Histological type	
Type I	455 (87.5)
Type II	65 (12.5)

Tumor diameter	
<2 cm	189 (36.3)
≥2 cm	331 (63.7)

Peritoneal cytology	
No	473 (91.0)
Yes	47 (9.0)

LVSI	
No	331 (74.4)
Yes	114 (25.6)

Lymph node involvement	
No	401 (77.1)
Yes	49 (9.4)
Unknown	70 (13.5)

Lymphadenectomy	
No	70 (13.5)
Yes	450 (86.5)

Adjuvant therapy	
No adjuvant therapy	285 (54.8)
Radiotherapy	10 (2.0)
Chemotherapy	165 (31.7)
Radiotherapy + cChemotherapy	60 (11.5)

Recurrence	
No	474 (91.2)
Yes	46 (8.8)

Follow-up (months)	
Median	53
Mean	48.7
Range	1–110

Data are expressed as *n* (%) or the means ± SD. FIGO = International Federation of Gynecology and Obstetrics; LVSI = lymphovascular space involvement.

**Table 2:** Multivariate Cox proportional hazards regression analysis for recurrence-free survival (RFS) in the training cohort.

Variables	Univariate analysis	*P* value	Multivariate analysis	*P* value
	OR (95% CI)		Adjusted OR (95% CI)	
Age (years)				
<60	1 (referent)			
≥60	2.4 (1.3–4.3)	0.003		

Depth of myometrial invasion				
<50%	1 (referent)			
≥50%	2.9 (1.6–5.2)	<0.001		

Cervical stromal invasion				
No	1 (referent)			
Yes	4.1 (2.2–7.6)	<0.001		

Histological grade				
Grade 1/2	1 (referent)		1 (referent)	
Grade 3	9.4 (5.0–18.0)	<0.001	4.2 (2.1–8.4)	<0.001

Histological type				
Type I	1 (referent)			
Type II	8.5 (4.7–15.2)	<0.001		

Tumor diameter				
<2 cm	1 (referent)		1 (referent)	
≥2 cm	6.6 (2.4–18.4)	<0.001	2.9 (1.0–8.4)	0.049

LVSI				
No	1 (referent)			
Yes	4.1 (2.2–7.5)	<0.001		

FIGO stage				
I	1 (referent)		1 (referent)	
II	5.4 (2.0–14.3)	0.001	2.4 (0.9–6.7)	0.099
III	8.8 (4.1–19.0)	<0.001	4.2 (1.9–9.5)	<0.001
IV	84.3 (36.3–195.5)	<0.001	15.1 (5.4–42.8)	<0.001

Peritoneal cytology				
No	1 (referent)		1 (referent)	
Yes	10.1 (5.6–18.3)	<0.001	2.5 (1.2–5.0)	0.013

Lymph node involvement				
No	1 (referent)			
Yes	8.1 (4.2–15.5)	<0.001		

Lymphadenectomy				
No	1 (referent)			
Yes	0.7 (0.3–1.4)	0.27		

Adjuvant therapy				
No adjuvant therapy	1 (referent)			
Radiotherapy	9.6 (2.0–45.6)	0.004		
Chemotherapy	6.7 (3.0–14.7)	<0.001		
Radiotherapy + chemotherapy	5.3 (2.0–14.1)	<0.001		

OR = odds ratio; CI = confidence interval; LVSI = lymphovascular space involvement; FIGO = International Federation of Gynecology and Obstetrics.

**Table 3:** The distribution of patients in the low- and high-risk cohorts estimated by the nomogram scores in the training cohort.

Variables	Low risk, *N* = 371 (71.3%)	High risk, *N* = 149 (28.7%)	*P* value
FIGO stage			<0.001
I	349 (94.1)	55 (36.9)	
II	18 (4.8)	19 (12.8)	
III	4 (1.1)	58 (38.9)	
IV	0 (0)	17 (11.4)	
Histological grade			<0.001
Grade 1/2	349 (94.1)	1 44 (29.5)	
Grade 3	22 (5.9)	105 (70.5)	
Primary tumor diameter			<0.001
<2 cm	179 (48.2)	10 (6.7)	
≥2 cm	192 (51.8)	139 (93.3)	
Peritoneal cytology			<0.001
No	370 (99.7)	103 (69.1)	
Yes	1 (0.3)	1 46 (30.9)	

Data are expressed as *n* (%). FIGO = International Federation of Gynecology and Obstetrics.

**Table 4:** Comparison of this study with previous studies of predictive models for recurrence-free survival of endometrial cancer patients.

	Obermair A	Bendifallah S	Ouldamer L	Wang J
Number of cases	2097	396	861	520
Recurrence-free survival	3-year	3-year	3-year	3- and 5-year
Histologic type	I, II	I	I, II	I, II
FIGO stage	I–III	I–III	I–III	I–IV
Factors				
Age	Yes	Yes	Yes	—
Histological grade	Yes	Yes	—	Yes
FIGO stage	Yes	—	Yes	Yes
Histologic type	Yes	—	Yes	—
Tumor diameter ≥ 2 cm	—	Yes	—	Yes
Myometrial invasion ≥ 50 %	—	Yes	—	—
LVSI	Yes	Yes	Yes	—
Peritoneal washing	Yes	—	—	Yes
Surgical nodal staging	—	—	Yes	
Comparison with FIGO stage	—	—	—	Superior
3-year internal validation	Yes (0.86)	Yes (0.74)	Yes (0.75)	Yes (0.89)
5-year internal validation	—	—	—	Yes (0.87)
3-year external validation	—	Yes (0.82)	—	Yes (0.88)
5-year external validation	—	—	—	Yes (0.87)

FIGO = International Federation of Gynecology and Obstetrics; LVSI = lymphovascular space involvement.

## Data Availability

The datasets used and/or analyzed during the current study are available from the corresponding author on reasonable request.

## Abbreviations

RFS: Recurrence-free survival
EC: Endometrial cancer
C-index: Concordance index
AUC: Area under the receiver operating characteristic curve
FIGO: Federation of Gynecology and Obstetrics
EC: Endometrial cancer
LVSI: Lymphovascular space involvement
HR: Hazard ratio.

## Supplementary Materials

Supplementary Materials Supplementary Table 1: clinicopathological characteristics of 520 endometrial cancer patients from the training cohort with or without recurrence. Supplementary Table 2: the clinicopathological characteristics of 445 endometrial cancer patients of the validation cohort. Supplementary Table 3: characteristics of 445 women with endometrial carcinoma in the validation cohort with or without recurrence. Supplementary Table 4: multivariate Cox proportional hazards regression analysis for recurrence-free survival (RFS) in the validation cohort.
