# Supplementary material for: Nomogram for Predicting Recurrence-Free Survival in Chinese Women with Endometrial Cancer after Initial Therapy: External Validation
Source: J Oncol. 2020 May 29;2020:2363545. doi: 10.1155/2020/2363545 (PMC7275963; doi:10.1155/2020/2363545)
Supplement: Supplementary Materials — Supplementary Table 1: clinicopathological characteristics of 520 endometrial cancer patients from the training cohort with or without recurrence. Supplementary Table 2: the clinicopathological characteristics of 445 endometrial cancer patients of the validation cohort. Supplementary Table 3: characteristics of 445 women with endometrial carcinoma in the validation cohort with or without recurrence. Supplementary Table 4: multivariate Cox proportional hazards regression analysis for recurrence-free survival (RFS) in the validation cohort. [file 2363545.f1.docx]

Supplemental data

S-Table 1. Clinicopathological characteristics of 520 endometrial cancer patients from the training cohort with or without recurrence

| Variables | Nonrecurrence  n=474 (91.2) | Recurrence  n=46 (8.8) | *P*-value |
| --- | --- | --- | --- |
| Age 0.004 | | | |
| <60 | 318 (67.1) | 21 (45.7) |  |
| ≥60 | 156 (32.9) | 25 (54.3) |  |
| Menopausal status <0.001 | | | |
| No | 172 (36.3) | 5 (10.9) |  |
| Yes | 302 (63.7) | 41 (89.1) |  |
| Depth of myometrial invasion <0.001 | | | |
| <50% | 367 (77.4) | 24 (52.2) |  |
| ≥50% | 107 (22.6) | 22 (47.8) |  |
| Cervical stromal invasion <0.001 | | | |
| No | 430 (90.7) | 31 (67.4) |  |
| Yes | 44 (9.3) | 15 (32.6) |  |
| Adnexal involvement <0.001 | | | |
| No | 460 (97.0) | 32 (69.6) |  |
| Yes | 14 (3.0) | 14 (30.4) |  |
| FIGO stage <0.001 | | | |
| I | 392 (82.7) | 12 (26.1) |  |
| II | 31 (6.5) | 6 (13.0) |  |
| III | 47 (9.9) | 15 (32.6) |  |
| IV | 4 (0.8) | 13 (28.3) |  |
| Histological type <0.001 | | | |
| Type I | 430 (90.7) | 25 (54.3) |  |
| Type II | 44 (9.3) | 21 (45.7) |  |
| Histological grade <0.001 | | | |
| G1/G2 | 380 (80.2) | 13 (28.3) |  |
| G3 | 94 (19.8) | 33 (71.7) |  |
| Primary tumor diameter <0.001 | | | |
| <2 | 185 (39.0) | 4 (8.7) |  |
| ≥2 | 289 (61.0) | 42 (91.3) |  |
| Peritoneal cytology <0.001 | | | |
| Absent | 446 (94.1) | 27 (58.7) |  |
| Present | 28 (5.9) | 1 19 (41.3) |  |
| LVSI <0.001 | | | |
| No | 409 (86.3) | 30 (65.2) |  |
| Yes | 65 (13.7) | 16 (34.8) |  |
| Lymph node involvement <0.001 | | | |
| No | 380(80.2) | 21(45.6) |  |
| Yes | 33(6.9) | 16(34.8) |  |
| Unknown | 61(12.9) | 9(19.6) |  |
| Lymphadenectomy 0.27 | | | |
| No | 61(12.9) | 9(19.6) |  |
| Yes | 413(87.1) | 37(80.4) |  |
| Adjuvant therapy <0.001 | | | |
| No adjuvant therapy | 277(58.4) | 8(17.4) |  |
| Radiotherapy | 8(1.7) | 2(4.3) |  |
| Chemotherapy | 137(28.9) | 28(60.9) |  |
| Radiotherapy+ Chemotherapy | 52(11.0) | 8(17.4) |  |

Data are expressed as n (%). FIGO= International Federation of Gynecology and Obstetrics; LVSI= lymphovascular space involvement.

S-Table 2. The clinicopathological characteristics of 445 endometrial cancer patients of the validation cohort

| Variables | Validation cohort |
| --- | --- |
|  | n=445 (No. of patients) % |
| Age (year) | |
| <60 | 310 (69.7) |
| ≥60 | 135 (30.3) |
| Menopausal status | |
| No | 143 (32.1) |
| Yes | 302 (67.9) |
| Depth of myometrial invasion | |
| <50% | 259 (58.2) |
| ≥50% | 186 (41.8) |
| Cervical stromal invasion | |
| No | 314 (70.6) |
| Yes | 131 (29.4) |
| Adnexal involvement | |
| No | 381 (85.6) |
| Yes | 64 (14.4) |
| FIGO stage | |
| I | 264 (59.3) |
| II | 38 (8.5) |
| III | 111(25.0) |
| IV | 32(7.2) |
| Histological grade | |
| Grade 1/2 | 297 (66.7) |
| Grade 3 | 148 (33.3) |
| Histological type | |
| Type I | 359 (80.7) |
| Type II | 86 (19.3) |
| Tumor diameter | |
| <2 | 303 (68.1) |
| ≥2 | 142 (31.9) |
| Peritoneal cytology | |
| No | 394 (88.5) |
| Yes | 51 (11.5) |
| LVSI | |
| No | 439 (84.4) |
| Yes | 81 (15.6) |
| Recurrence | |
| No | 353 (79.3) |
| Yes | 92 (20.7) |
| Follow-up (months) | |
| Median | 28 |
| Mean | 32.3±21.9 |
| Range | 1-112 |

Data are expressed as n (%) or the mean ± SD. Abbreviations: FIGO= International Federation of Gynecology and Obstetrics; LVSI=lymphovascular space involvement.

S-Table 3. Characteristics of 445 women with endometrial carcinoma in the validation cohort with or without recurrence

| Variables | Nonrecurrence  (No. of patients) %  n=353 (79.3) | Recurrence  (No. of patients) %  n=92 (20.7) | *P*-value |
| --- | --- | --- | --- |
| Age 0.071 | | | |
| <60 | 253 (71.7) | 57 (62.0) |  |
| ≥60 | 100 (28.3) | 35 (38.0) |  |
| Menopausal status 0.017 | | | |
| No | 123 (34.8) | 20 (21.7) |  |
| Yes | 230 (65.2) | 72 (78.3) |  |
| Depth of myometrial invasion <0.001 | | | |
| <50% | 227 (64.3) | 32 (34.8) |  |
| ≥50% | 126 (35.7) | 60 (65.2) |  |
| Cervical stromal invasion <0.001 | | | |
| No | 272 (77.1) | 42 (45.7) |  |
| Yes | 81 (22.9) | 50 (54.3) |  |
| Adnexal involvement <0.001 | | | |
| No | 322 (91.2) | 59 (64.1) |  |
| Yes | 31 (8.8) | 33 (35.9) |  |
| FIGO stage | | <0.001 | |
| I | 249 (70.5%) | 15 (16.3) |  |
| II | 33 (9.3) | 5 (5.4) |  |
| III | 65 (18.4) | 46 (50.0) |  |
| IV | 6 (1.7) | 26 (28.3) |  |
| Histological type | | <0.001 | |
| Type I | 298 (84.4) | 61 (66.3) |  |
| Type II | 55 (15.6) | 31 (33.7) |  |
| Histological grade <0.001 | | | |
| G1/G2 | 263 (74.5) | 1 34 (37.0) |  |
| G3 | 90 (25.5) | 58 (63.0) |  |
| Primary tumor diameter <0.001 | | | |
| <2 | 273(77.3) | 30 (32.6) |  |
| ≥2 | 80 (22.7) | 62 (67.4) |  |
| Peritoneal cytology <0.001 | | | |
| Absent | 327 (92.6) | 67 (72.8) |  |
| Present | 26 (7.4) | 1 25 (27.2) |  |
| LVSI <0.001 | | | |
| No | 285 (80.7) | 46 (50.0) |  |
| Yes | 68 (19.3) | 46 (50.0) |  |

Data are expressed as n (%). FIGO= International Federation of Gynecology and Obstetrics; LVSI= lymphovascular space involvement.

S-Table 4. Multivariate Cox proportional hazards regression analysis for recurrence-free survival (RFS) in the validation cohort

|  | Univariate analysis |  | Multivariate analysis | |  |
| --- | --- | --- | --- | --- | --- |
| Variables | Adjusted HR  (95% CI) | P-value | Adjusted HR  (95% CI) | P-value | |
| FIGO stage | | | | | |
| I | 1 (Referent) |  | 1 (Referent) |  | |
| II | 2.0 (0.7-5.5) | 0.18 | 1.0 (0.3-2.9) |  | |
| III | 7.3(4.1-13.0) | <0.001 | 3.3 (1.7-6.3) | <0.001 | |
| IV | 26.2(13.8-49.7) | <0.001 | 8.0 (3.7-17.1) | <0.001 | |
| Histological grade | | | | | |
| G1/G2 | 1 (Referent) |  | 1 (Referent) |  | |
| G3 | 3.7(2.4-5.7) | <0.001 | 2.1 (1.3-3.3) | 0.001 | |
| Tumor diameter | | | | | |
| <2 | 1 (Referent) |  | 1 (Referent) |  | |
| ≥2 | 5.9(3.8-9.4) | <0.001 | 3.0 (1.8-4.9) | 0.049 | |
| Peritoneal cytology | | | | | |
| No | 1 (Referent) |  | 1 (Referent) |  | |
| Yes | 4.4(2.8-7.0) | <0.001 | 1.7 (1.1-2.9) | 0.029 | |
| Cervical stromal invasion | | | | | |
| No | 1 (Referent) |  | 1 (Referent) |  | |
| Yes | 2.9(1.9-4.3) | <0.001 | 2.1（1.3-3.3） | 0.002 | |

Abbreviations: OR=odds ratio; CI=confidence interval; LVSI=lymphovascular space involvement; FIGO=International Federation of Gynecology and Obstetrics.
